# Enhanced Open-Circuit Voltage in Perovskite Solar Cells with Open-Cage [60]Fullerene Derivatives as Electron-Transporting Materials

**DOI:** 10.3390/ma12081314

**Published:** 2019-04-23

**Authors:** Edison Castro, Albert Artigas, Anna Pla-Quintana, Anna Roglans, Fang Liu, Frank Perez, Agustí Lledó, X.-Y. Zhu, Luis Echegoyen

**Affiliations:** 1 Department of Chemistry, University of Texas at El Paso El Paso, TX 79968, USA; faperez2@miners.utep.edu; 2 Institut de Química Computacional i Catàlisi (IQCC), Department de Química, Universitat de Girona, 17003 Girona, Catalonia Spain; albert.artigas@udg.edu (A.A.); anna.plaq@udg.edu (A.P.-Q.); anna.roglans@udg.edu (A.R.); agusti.lledo@udg.edu (A.L.); 3 Department of Chemistry, Columbia University, New York, NY 10027, USA; fl2454@columbia.edu (F.L.); xz2324@columbia.edu (X.-Y.Z.)

**Keywords:** open-cage fullerenes, perovskite solar cells, improving open circuit voltage

## Abstract

The synthesis, characterization, and incorporation of open-cage [60]fullerene derivatives as electron-transporting materials (ETMs) in perovskite solar cells (PSCs) with an inverted planar (p-i-n) structure is reported. Following optical and electrochemical characterization of the open-cage fullerenes **2a**–**c**, p-i-n PSCs with a indium tin oxide (ITO)/poly(3,4-ethylenedioxythiophene)-polystyrene sulfonate (PEDOT:PSS)/perovskite/fullerene/Ag structure were prepared. The devices obtained from **2a**–**b** exhibit competitive power conversion efficiencies (PCEs) and improved open-circuit voltage (*V_oc_*) values (>1.0 V) in comparison to a reference cell based on phenyl-C_61_-butyric-acid methyl-ester (PC_61_BM). These results are rationalized in terms of a) the higher passivation ability of the open-cage fullerenes with respect to the other fullerenes, and b) a good overlap between the highest occupied molecular orbital/lowest unoccupied molecular orbital (HOMO/LUMO) levels of **2a**–**b** and the conduction band of the perovskite.

## 1. Introduction

Perovskite solar cells (PSCs) are an emerging class of photovoltaic devices, which promise to rival the performance of state-of-the-art cells, with current record power conversion efficiencies (PCEs) recently reaching 24.2% [1] A major advantage of PSCs is their facile manufacturing process, which is mostly based on solution processing. However, a number of challenges need to be addressed before a marketable technology is available, including (a) cell performance, (b) cell stability, and (c) upscaling beyond laboratory scale toward the industrial production of commercially viable photovoltaic devices [2,3,4,5,6,7]. Another desirable yet unmet objective is the replacement of Pb by less toxic metals in the perovskite structure [8]. 

**Figure 1:**
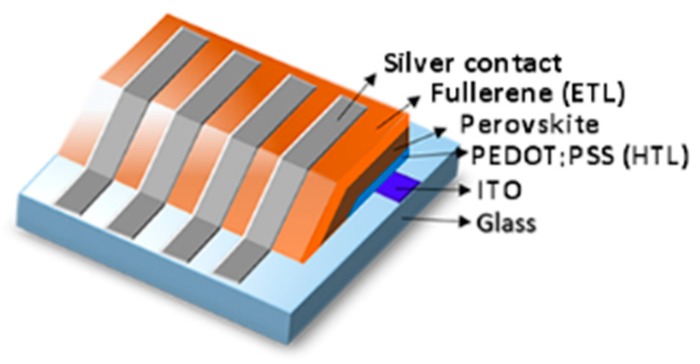
Inverted planar perovskite solar cell (PSC) representation with the structure of indium tin oxide (ITO)/poly(3,4-ethylenedioxythiophene)-polystyrene sulfonate (PEDOT:PSS)/perovskite/electron-transport layer (ETL)/silver.

A PSC consists of a sandwiched structure containing a transparent conductive oxide, a hole transport layer (HTL), a perovskite photo-absorber layer, an electron-transport layer (ETL), and a back-contact electrode [9,10,11,12,13,14,15]. Among the various cell configurations available for PSCs, the inverted planar structure (p-i-n) (Figure 1) is the most attractive in terms of manufacturing, because the ETL, which is typically a fullerene derivative, is solution-processed [16], as opposed to the metal oxides employed in the regular planar and mesoscopic configurations, which require high-temperature annealing steps [17,18,19]. Importantly, p-i-n PSCs can be easily integrated in flexible devices [20]. A downside of the p-i-n configuration is that high open circuit voltage (*V_oc_*) values are difficult to achieve. Successful strategies to overcome this limitation rely on the incorporation of dopant materials [21,22,23], interfacial engineering [24,25,26], morphology control [27,28], or the replacement of the HTL [29,30].

On the other hand, the replacement of phenyl-C_61_-butyric-acid methyl-ester (PC_61_BM) by other fullerenes as the ETL has found limited success, with only a few reports of *V_oc_* values beyond the 1.0 V threshold [31,32]. For the specific case of poly(3,4-ethylenedioxythiophene)-polystyrene sulfonate (PEDOT:PSS)-based devices, examples are even scarcer [11,33,34]. The development of PSCs with PC_61_BM surrogates relies mostly on the incorporation of highly crystalline ETLs. The resulting cells benefit from reduced energy disorder and improved charge trap passivation [35,36]. While this approach has been implemented with a variety of fullerenes, the vast majority of them consist of [3+2], [4+2], or cyclopropane adducts [15]. Thus, the introduction of structurally novel fullerene scaffolds that can expand the ETL repertoire is highly desirable. 

Overall, the highest certified PCE value reported for PSCs with p-i-n configurations is 20.9% [37]. The development of efficient ETMs that can increase the efficiency of PSCs without the need of additives or complex manufacturing techniques is a major challenge in the field of inverted planar PSCs.

**Scheme 1:**
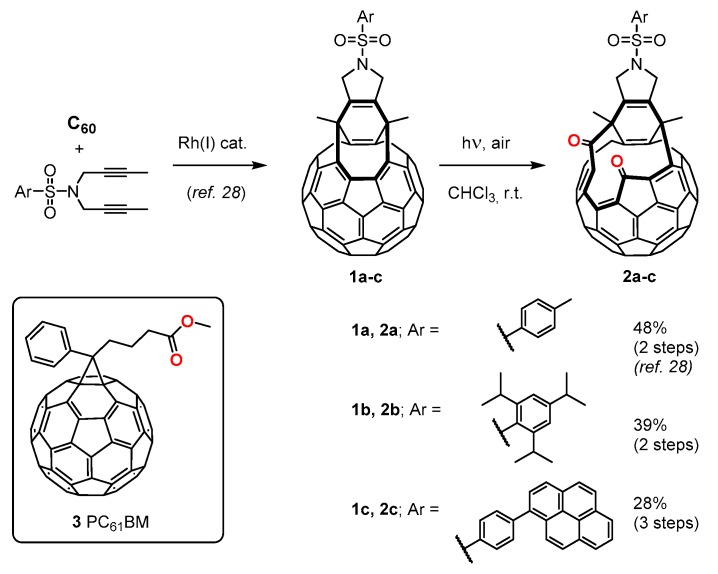
Synthesis of open-cage fullerene derivatives **2a**–**c**, and (inset) reference compound used in this study ((**3**, PC_61_BM), See Supporting Information for the synthesis of **1c**).

Open-cage fullerenes are a family of synthetic derivatives in which the three-dimensional backbone of the fullerene cage is distorted by the scission of one or more C–C bonds. Open-cage fullerenes have been successfully used as electron-accepting materials or as additives in bulk-heterojunction solar cells [38,39,40]. However, their use as ETLs in PSCs remains unexplored. Some of us recently reported a straightforward methodology for the synthesis of open-cage fullerene derivatives (Scheme 1) [41,42]. The promising electrochemical properties and remarkable stability of these compounds, together with the fact that fullerene derivatives are so far the materials of choice as the ETL for p-i-n PSCs, prompted us to study the use of these compounds for PSCs.

Herein, we report for the first time the incorporation of open-cage fullerenes in p-i-n PSCs. The cells have been thoroughly characterized, and their photovoltaic performance has been studied. We demonstrate that open-cage fullerenes exhibit improved performances with respect to PC_61_BM-based devices.

## 2. Results

The open-cage derivatives used in this study, **2a**–**c**, were prepared by the photochemical oxidation of bis(fulleroids) **1a**–**c**, which can be obtained from [60]fullerene in one step using our Rh(I)-catalyzed cycloaddition protocol (Scheme 1) [41]. Importantly, unlike precursors **1a**-**c**, open-cage derivatives **2a**–**c** do not suffer further degradation upon exposure to light and air. In addition to the parent open-cage derivative **2a**, we selected compounds possessing desirable features for PSC manufacturing, such as the improved solubility of **2b** or the light-harvesting ability of **2c**. With **2a**–**c** in hand, we first assessed their optical and electrochemical properties. In solution, **2a**–**c** display remarkable absorption maxima in the visible region (λ_max_ = 705–710 nm, Figure S12). The electrochemical properties of **2a**–**c** (Figure S13) and PC_61_BM (**3**) were determined by cyclic voltammetry (CV) in ortho-dichlorobenzene (*o*-DCB) (see the Supporting Information). 

Compounds **2a**–**c** exhibit three fully reversible cathodic electrochemical behaviors between −0.8 and −2.3 V at a scan rate of 100 mV s^−1^. The highest occupied molecular orbital/lowest unoccupied molecular orbital (HOMO/LUMO) values were estimated from the ultraviolet (UV) and CV measurements [43].

The optical properties of compounds **2a**–**c** are summarized in Table 1. Overall, the photophysical and electrochemical properties of the open-cage fullerenes **2a–c** are very similar to those of PC_61_BM, even though the C_60_ cage skeleton is significantly altered with respect to the latter. These results encouraged us to incorporate **2a**–**c** as the electron-transporting materials (ETMs) in PSCs.

**Figure 2:**
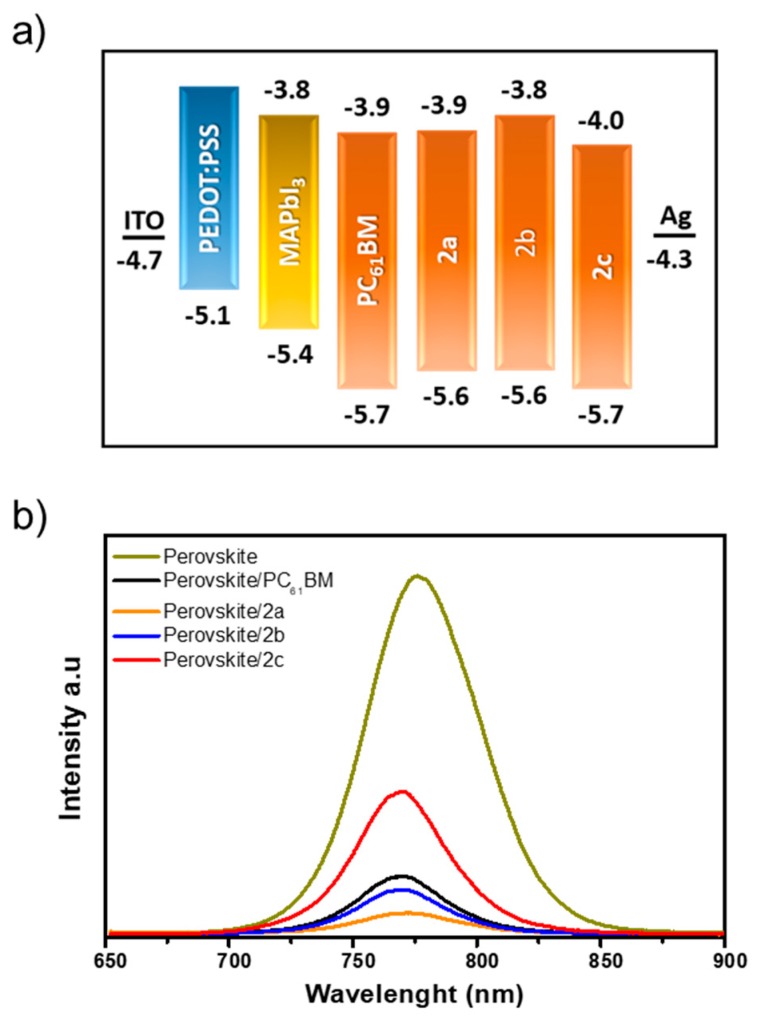
(**a**) Schematic illustration of the estimated HOMO and LUMO energy levels, estimated from cyclic voltammetry (CV) and UV-vis. (**b**) Steady-state photoluminescence (PL) spectra of the perovskite and perovskite/ETM films.

Figure 2a shows the energy level diagram estimated from the onset potential of the first reductions and the maximum onset absorption from UV-vis spectra for all the compounds [44]. The electrical conductivities for PC_61_BM and compounds **2a**–**c** films were compared by recording current–voltage (*J-V*) curves for electron-only devices with a structure of ITO/Al/ETM/Al. All the ETLs showed similar electron conductivities (4.8, 3.5, 2.8 and 3.7 × 10^−4^ cm^2^ V^−1^·s^−1^ for **2a**, **2b**, **2c**, and PC_61_BM, respectively).

To probe the passivation ability of compounds **2a**–**c**, we studied the photoluminescence (PL) and time-resolved (TR) PL of the photoactive layer (perovskite) with and without **2a**–**c**, using PC_61_BM as the control (Figure 2b and Figure S14). A significant PL quenching effect was observed for the perovskite layer coated with the open-cage fullerenes **2a**, **2b**, and PC_61_BM (Figure 2b). Meanwhile, the PL intensity of the perovskite increases when using **2c**, which is an effect that can be attributed to the lower solubility of **2c** in chlorobenzene (CB) (Figure S15). Compound **2a** exhibits a higher passivation ability than the other fullerenes, resulting in a more pronounced inhibition of the electron–hole recombination processes [45].

Figure S14 shows the TR-PL decay measurements, monitoring the emission peak of PC_61_BM and **2a**–**c** coated perovskite layers as a function of time. The pristine perovskite layer exhibits a PL lifetime of about 25.6 ns, whereas perovskite/**2a**, perovskite/**2b**, perovskite/**2c**, and perovskite/PC_61_BM exhibit PL lifetimes of 3.8 ns, 4.7 ns, 9.2 ns, and 14.1 ns, respectively. Faster decays are measured for the samples coated with **2a** and **2b**, indicating that the charge transfer processes are faster than the charge recombination in the perovskite layer [46].

Compounds **2a**–**c** were incorporated in PSCs with an ITO/PEDOT:PSS/perovskite/fullerene/Ag structure (Figure 1, see Supporting Information for details). The **2a** (1.01 V) and **2b** (0.97 V)-based devices showed a significant enhancement of *V_oc_* values compared to the PC_61_BM (0.92 V)-based devices. 

**Figure 3:**
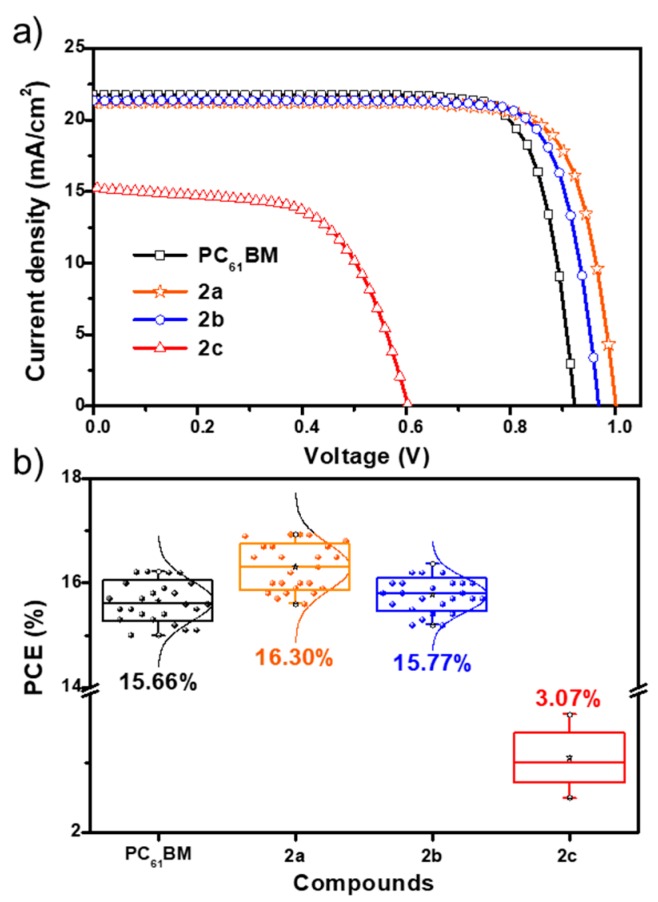
(**a**) Current–voltage (*J-V*) curves under 1 sun of illumination (100 mW/cm^2^) in forward voltage scans. (**b**) The power conversion efficiency (PCE) histograms measured for 25 independent cells.

On the other hand, the lower solubility of **2c** led to low-quality films, resulting in lower photovoltaic performances for the **2c**-based devices (Figure 3). Table 2 summarizes the performances of the PSC devices incorporating PC_61_BM and compounds **2a**–**c**. The work functions of the charge transport materials affect the *V_oc_* of PSCs significantly, so the higher *V_oc_* values obtained from **2a** and **2b**-based devices can be attributed to their higher LUMO values, when compared with PC_61_BM [25,26,46,47]. Commonly, the *V_oc_* values are improved by inserting a work-function interlayer between the perovskite and the ETL [26].

PSCs based on all the fullerene derivatives showed negligible hysteretic behavior (Figure S16). Device performance reproducibilities were calculated from the PCE distributions measured for 25 independent cells (Figure 3b). Figure S17 shows the external quantum efficiency (EQE) of the PSCs based on PC_61_BM and **2a**–**c**; the devices based on **2a** show higher photoresponse around 600 nm and 750 nm. The integrated photocurrent densities based on EQE measurements (Figure S17) are consistent with those from *J-V* measurements (Table 2). PC_61_BM devices exhibited a PCE value of 16.22% with a *V_oc_* value of 0.92 V, a short circuit current (*J_sc_*) value of 21.77 mA·cm^−2^, and a fill factor (FF) value of 0.80. In contrast, **2a** devices exhibited a PCE value of 16.92% with a *V_oc_* value of 1.01 V, a *J_sc_* value of 21.21 mA·cm^−2^, and a FF value of 0.79. The improved device performance was attributed to the better passivation ability of compounds **2a** and **2b**, because of their higher work function, which matches well with the conduction band of the perovskite [25,26,46,47].

The stabilities of PSCs fabricated with PC_61_BM, **2a**, and **2b** were monitored under ca. 25% humidity in air at room temperature without encapsulation for 10 days. The normalized PCEs against time are shown in Figure S18. PC_61_BM-based devices lost 67% of their initial PCE; this was similar to the PC_61_BM-based devices **2a** and **2b**-based devices, which lost 65% and 63% of their initial PCE, respectively. Meanwhile, the devices based on **2c** lost 71% of their initial PCE. Thus, the open-cage compounds are comparable to PC_61_BM in terms of cell stability.

## 3. Conclusions

In conclusion, we have successfully prepared a series of p-i-n type PSCs incorporating dicarbonylic open-cage [60]fullerene derivatives **2a**–**c** as the ETL. For those compounds with appropriate solubility, the resulting PSCs offer performances rivaling or even superior to those of analogous cells employing the PC_61_BM reference. These results are commensurate with a good overlap between the HOMO/LUMO levels of fulleroids **2a**–**b** and the conduction band of the perovskite. The modularity of our synthetic approach to open-cage fullerene derivatives **2a**–**c** offers a promising opportunity to develop superior PSCs beyond this preliminary account.

## Figures and Tables

**Table 1:** Optical bandgap, onset reduction, and lowest unoccupied molecular orbital/highest occupied molecular orbital (LUMO/HOMO) energy levels of the open-cage **2a**–**c** and compound **3**.

Comp	λ_max_ (nm)	E_g_ (ev)	E_red_ (V)	LUMO (ev)	HOMO (ev)
**2a**	709	1.75	0.94	−3.86	−5.61
**2b**	705	1.76	0.99	−3.81	−5.57
**2c**	705	1.76	0.85	−3.95	−5.71
**PC_61_BM**	718	1.73	0.87	−3.93	−5.66

**Table 2:** Summary of device performance. The calculated short circuit current (*J_sc_*) values were obtained from the external quantum efficiency (EQE) curves. Values in parentheses represent the best values measured, ^a^ are the average values, and * are the calculated values. PC_61_BM: phenyl-C_61_-butyric-acid methyl-ester.

Compound	J_sc_* (mA cm^−2^)	*J_sc_* (mA cm^−2^)	*V_oc_* (V)	FF (%)	PCE (%)
**2a**	21.05	21.17 ^a^ (21.21)	0.99 ^a^ (1.01)	0.79	16.30 ± 0.62 (16.92)
**2b**	21.11	20.98 ^a^ (21.37)	0.96 ^a^ (0.97)	0.79	15.77 ± 0.60 (16.37)
**2c**	14.87	15.01 ^a^ (15.20)	0.53 ^a^ (0.60)	0.41	3.07 ± 0.67 (3.74)
**PC_61_BM**	21.22	21. 56 ^a^ (21.77)	0.88 ^a^ (0.92)	0.80	15.66 ± 0.56 (16.22)

## Supplementary Materials

The following are available online at https://www.mdpi.com/1996-1944/12/8/1314/s1, Figure S-1. ^1^H NMR (400 MHz, CDCl_3_) of compound **3a**. Figure S-2. ^1^H NMR (400 MHz, CDCl_3_) of compound **3b**. Figure S-3. ^1^H NMR (400 MHz, CDCl_3_) of compound **3c**. Figure S-4. ^1^H NMR (400 MHz, CDCl_3_) of compound **1a**. Figure S-5. ^1^H NMR (400 MHz, CDCl_3_) of compound **1b**. Figure S-6. ^1^H NMR (400 MHz, o-DCB-*_d4_*/CS_2_) of compound S-5. Figure S-7. ^1^H NMR (400 MHz, CDCl_3_) of compound **1c**. Figure S-8. ^1^H NMR (400 MHz, CDCl_3_) of compound **2a**. Figure S-9. ^1^H NMR (400 MHz, CDCl_3_) of compound **2b**. Figure S-10. ^13^C NMR (100 MHz, CDCl_3_) of compound **2b**. Figure S-11. ^1^H NMR (400 MHz, CDCl_3_) of compound **2c**. Figure S-12. UV-vis spectra of compounds **2a-c** and PC_61_BM. Figure S-13. Cyclic voltammetry of compounds **2a-c**. Figure S-14. Time-resolved photoluminescence of perovskite, perovskite/compounds **2a-c** and perovskite/PC_61_BM films. Figure S-15. Fullerene derivatives **2a-c** and PC_61_BM in *o*-dichlorobezene (20 mg/mL). Figure S-16. *J*-*V* curves of the inverted PSCs based on PC_61_BM (a) and **2a,b** (b and c, respectively) with respect to forward and reverse scan directions (the scanning rate was 100 mV/s). Figure S-17. EQE measurements for **2a-c** and PC_61_BM-based devices. Figure S-18. Stability studies of **2a-c** and PC_61_BM-based devices. Figure S-19. Top-view SEM image of the perovskite film.

## References

[1] NREL Best Research-Cell Efficiencies. https://www.nrel.gov/pv/assets/pdfs/best-research-cell-efficiencies-190416.pdf.

[2] Rong Y., Hu Y., Mei A., Tan H., Saidaminov M.I., Seok S.I., McGehee M.D., Sargent E.H., Han H. (2018). Challenges for commercializing perovskite solar cells. Science.

[3] Li Z., Klein T.R., Kim D.H., Yang M., Berry J.J., van Hest M.F.A.M., Zhu K. (2018). Scalable fabrication of perovskite solar cells. Nat. Rev. Mater..

[4] Galagan Y. (2018). Perovskite Solar Cells: Toward Industrial-Scale Methods. J. Phys. Chem. Lett..

[5] Wang F., Cao Y., Chen C., Chen Q., Wu X., Li X., Qin T., Huang W. (2018). Materials toward the upscaling of perovskite solar cells: progress, challenges, and strategies. Adv. Funct. Mater..

[6] Abate A., Correa-Baena J.-P., Saliba M., Su’ait M.S., Bella F. (2018). Perovskite Solar Cells: From the laboratory to the assembly line. Chem. Eur. J..

[7] Bella F., Renzi P., Cavallo C., Gerbaldi C. (2018). Caesium for perovskite solar cells: An overview. Chem. Eur. J..

[8] Giustino F., Snaith H.J. (2016). Toward Lead-free perovskite solar cells. ACS Energy Lett..

[9] Castro E., Fernandez-Delgado O., Arslan F., Zavala G., Yang T., Seetharaman S., Dsouza F., Echegoyen L. (2018). New thiophene-based C60 fullerene derivatives as efficient electron transporting materials for perovskite solar cells. New J. Chem..

[10] Castro E., Sisto T.J., Romero E.L., Liu F., Peurifoy S.R., Wang J., Zhu X., Nuckolls C., Echegoyen L. (2017). Cove-Edge Nanoribbon materials for efficient inverted halide perovskite solar cells. Angew. Chem. Int. Ed..

[11] Castro E., Zavala G., Seetharaman S., D’Souza F., Echegoyen L. (2017). Impact of fullerene derivative isomeric purity on the performance of inverted planar perovskite solar cells. J. Mater. Chem. A..

[12] Peurifoy S.R., Castro E., Liu F., Zhu X.Y., Ng F., Jockusch S., Steigerwald M.L., Echegoyen L., Nuckolls C., Sisto T.J. (2018). Three-dimensional graphene nanostructures. J. Am. Chem. Soc..

[13] Tian C., Castro E., Betancourt-Solis G., Nan Z.-A., Fernandez-Delgado O., Jankuru S., Echegoyen L. (2018). Fullerene derivative with a branched alkyl chain exhibits enhanced charge extraction and stability in inverted planar perovskite solar cells. New J. Chem..

[14] Tian C., Castro E., Wang T., Betancourt-Solis G., Rodriguez G., Echegoyen L. (2016). Improved performance and stability of inverted planar perovskite solar cells using fulleropyrrolidine layers. ACS Appl. Mater. Interfaces..

[15] Tian C., Kochiss K., Castro E., Betancourt-Solis G., Han H., Echegoyen L. (2017). A dimeric fullerene derivative for efficient inverted planar perovskite solar cells with improved stability. J. Mater. Chem. A..

[16] Khadka D.B., Shirai Y., Yanagida M., Miyano K. (2019). Unraveling the impacts induced by organic and inorganic hole transport layers in inverted halide perovskite solar cells. ACS Appl. Mater. Interfaces..

[17] Castro E., Murillo J., Fernandez-Delgado O., Echegoyen L. (2018). Progress in fullerene-based hybrid perovskite solar cells. J. Mater. Chem. C..

[18] Fang Y., Bi C., Wang D., Huang J. (2017). The functions of fullerenes in hybrid perovskite solar cells. ACS Energy Lett..

[19] Liu T., Chen K., Hu Q., Zhu R., Gong Q. (2016). Inverted perovskite solar cells: Progresses and perspectives. Adv. Energy Mater..

[20] Wang Y.-C., Li X., Zhu L., Liu X., Zhang W., Fang J. (2017). Efficient and hysteresis-free perovskite solar cells based on a solution processable polar fullerene electron transport layer. Adv. Energy Mater..

[21] Xia X., Jiang Y., Wan Q., Wang X., Wang L., Li F. (2018). Lithium and silver co-doped nickel oxide hole-transporting layer boosting the efficiency and stability of inverted planar perovskite solar cells. ACS Appl. Mater. Interfaces.

[22] Wang Y., Wang S., Chen X., Li Z., Wang J., Li T., Deng X. (2018). Largely enhanced VOC and stability in perovskite solar cells with modified energy match by coupled 2D interlayers. J. Mater. Chem. A..

[23] Chen W., Zhou Y., Wang L., Wu Y., Tu B., Yu B., Liu F., Tam H.-W., Wang G., Djurišić A.B., Huang L., He Z. (2018). Molecule-doped nickel oxide: Verified charge transfer and planar inverted mixed cation perovskite solar cell. Adv. Mater..

[24] Chen J., Lian X., Zhang Y., Yang W., Li J., Qin M., Lu X., Wu G., Chen H. (2018). Interfacial engineering enables high efficiency with a high open-circuit voltage above 1.23 V in 2D perovskite solar cells. J. Mater. Chem. A..

[25] Xue Q., Bai Y., Liu M., Xia R., Hu Z., Chen Z., Jiang X.-F., Huang F., Yang S., Matsuo Y., Yip H.-L., Cao Y. (2017). Dual interfacial modifications enable high performance semitransparent perovskite solar cells with large open circuit voltage and fill factor. Adv. Energy Mater..

[26] Bai Y., Yu H., Zhu Z., Jiang K., Zhang T., Zhao N., Yang S., Yan H. (2015). High performance inverted structure perovskite solar cells based on a PCBM:polystyrene blend electron transport layer. J. Mater. Chem. A..

[27] Cheng J., Zhang H., Zhang S., Ouyang D., Huang Z., Nazeeruddin M.K., Hou J., Choy W.C.H. (2018). Highly efficient planar perovskite solar cells achieved by simultaneous defect engineering and formation kinetic control. J. Mater. Chem. A..

[28] Li W., Wu X., Qin H., Zhao Z., Liu H. (2016). Light-Driven and Light-Guided Microswimmers. Adv. Funct. Mater..

[29] Zhang H., Wang H., Zhu H., Chueh C.-C., Chen W., Yang S., Jen A.K.-Y. (2018). Low-temperature solution-processed CuCrO_2_ hole-transporting layer for efficient and photostable perovskite solar cells. Adv. Energy Mater..

[30] Wang H., Yu Z., Lai J., Song X., Yang X., Hagfeldt A., Sun L. (2018). One plus one greater than two: High-performance inverted planar perovskite solar cells based on a composite CuI/CuSCN hole-transporting layer. J. Mater. Chem. A..

[31] Yan K., Chen J., Ju H., Ding F., Chen H., Li C.-Z. (2018). Achieving high-performance thick-film perovskite solar cells with electron transporting Bingel fullerenes. J. Mater. Chem. A..

[32] Meng X., Bai Y., Xiao S., Zhang T., Hu C., Yang Y., Zheng X., Yang S. (2016). Designing new fullerene derivatives as electron transporting materials for efficient perovskite solar cells with improved moisture resistance. Nano Energy..

[33] Gil-Escrig L., Momblona C., Sessolo M., Bolink H.J. (2016). Fullerene imposed high open-circuit voltage in efficient perovskite based solar cells. J. Mater. Chem. A..

[34] Chiang C.-H., Tseng Z.-L., Wu C.-G. (2014). Planar heterojunction perovskite/PC71BM solar cells with enhanced open-circuit voltage via a (2/1)-step spin-coating process. J. Mater. Chem. A..

[35] Xie F., Zhang L., Su D., Jaroniec M., Qiao S.-Z. (2017). Na_2_Ti_3_O_7_@N-Doped carbon hollow spheres for sodium-ion batteries with excellent rate performance. Adv. Mater..

[36] Khadka D.B., Shirai Y., Yanagida M., Noda T., Miyano K. (2018). Tailoring the open-circuit voltage deficit of wide-band-gap perovskite solar cells using alkyl chain-substituted fullerene derivatives. ACS Appl. Mater. interfaces..

[37] Luo D., Yang W., Wang Z., Sadhanala A., Hu Q., Su R., Shivanna R., Trindade G.F., Watts J.F., Xu Z. (2018). Enhanced photovoltage for inverted planar heterojunction perovskite solar cells. Science.

[38] Chen C.-P., Huang C.-Y., Chuang S.-C. (2015). Highly thermal stable and efficient organic photovoltaic cells with crosslinked networks appending open-cage fullerenes as additives. Adv. Funct. Mater..

[39] Murata M., Morinaka Y., Murata Y., Yoshikawa O., Sagawa T., Yoshikawa S. (2011). Modification of the σ-framework of [60]fullerene for bulk-heterojunction solar cells. Chem. Commun..

[40] Chen C.-P., Lin Y.-W., Horng J.-C., Chuang S.-C. (2011). Open-cage fullerenes as n-type materials in organic photovoltaics: Relevance of frontier energy levels, carrier mobility and morphology of different sizable open-cage fullerenes with power conversion efficiency in devices. Adv. Energy Mater..

[41] Artigas A., Pla-Quintana A., Lledó A., Roglans A., Solà M. (2018). Expeditious preparation of open-cage fullerenes by Rhodium(I)-catalyzed [2+2+2] cycloaddition of diynes and C60: An experimental and theoretical study. Chem. Eur. J..

[42] Artigas A., Lledó A., Pla-Quintana A., Roglans A., Solà M. (2017). Cover feature: A computational study of the intermolecular [2+2+2] cycloaddition of acetylene and C60 catalyzed by wilkinson’s catalyst. Chem. Eur. J..

[43] Closs G.L., Gautam P., Zhang D., Krusic P.J., Hill S.A., Wasserman E. (1992). Steady-state and time-resolved direct detection EPR spectra of fullerene triplets in liquid solution and glassy matrixes: evidence for a dynamic Jahn-Teller effect in triplet C60. J. Phys. Chem..

[44] Sun Q.J., Wang H.Q., Yang C.H., Li Y.F. (2003). Synthesis and electroluminescence of novel copolymers containing crown ether spacers. J. Mater. Chem..

[45] Liang P.-W., Liao C.-Y., Chueh C.-C., Zuo F., Williams S.T., Xin X.-K., Lin J., Jen A.K.Y. (2014). Additive enhanced crystallization of solution-processed perovskite for highly efficient planar-heterojunction solar cells. Adv. Mater..

[46] Wu C.-G., Chiang C.-H., Chang S.H. (2016). A perovskite cell with a record-high-Voc of 1.61 V based on solvent annealed CH_3_NH_3_PbBr_3_/ICBA active layer. Nanoscale.

[47] Chen S., Hou Y., Chen H., Richter M., Guo F., Kahmann S., Tang X., Stubhan T., Zhang H., Li N. (2016). Exploring the limiting open-circuit voltage and the voltage loss mechanism in planar ch3nh3pbbr3 perovskite solar cells. Adv. Energy Mater..

