# Supplementary material for: Enhanced Open-Circuit Voltage in Perovskite Solar Cells with Open-Cage [60]Fullerene Derivatives as Electron-Transporting Materials
Source: Materials (Basel). 2019 Apr 23;12(8):1314. doi: 10.3390/ma12081314 (PMC6515431; doi:10.3390/ma12081314)
Supplement: Supplementary file 1 [file materials-12-01314-s001.pdf]

materials

MDPI

Communication

Enhanced Open-Circuit Voltage in Perovskite Solar Cells with Open-Cage [60]Fullerene Derivatives as Electron-Transporting Materials

Edison Castro [1,†,*], Albert Artigas [2,†], Anna Pla-Quintana [2], Anna Roglans [2], Fang Liu [3], Frank Perez [1], Agustí Lledó [2], X-Y. Zhu [3] and Luis Echegoyen [1,*]

[1] Department of Chemistry, University of Texas at El Paso El Paso, TX 79968, USA; faperez2@miners.utep.edu (F.P.)
[2] Institut de Química Computacional i Catàlisi (IQCC), Department de Química, Universitat de Girona, Girona 17003, Catalonia Spain; albert.artigas@udg.edu (A.A.); anna.plaq@udg.edu (A.P.-Q.); anna.roglans@udg.edu (A.R.); agusti.lledo@udg.edu (A.L.)
[3] Department of Chemistry, Columbia University, New York, New York 10027, USA; fl2454@columbia.edu (F.L.); xz2324@columbia.edu (X.Y.Z.)
* Correspondence: edisoncastro2004@hotmail.com (E.C.); echegoyen@utep.edu (L.E.)
† These authors contributed equally.

Materials and methods

All chemicals were reagent grade, purchased from Sigma Aldrich. Silica gel (Redisep silica, 40-60 µ, 60 Å) was used to separate the products from the pristine fullerene. MALDI-TOF mass spectrometric measurements were conducted on a Bruker Microflex LRF mass spectrometer on reflector positive mode. NMR spectra were recorded using a Bruker 400 MHz spectrometer. The UV/Vis-NIR spectra were taken using a Cary 5000 UV/Vis-NIR spectrophotometer using toluene or carbon disulfide solutions. Cyclic voltammetry (CV) was carried out under an argon atmosphere at room temperature using a CH Instrument Potentiostat. Scan rate for CV experiments was 100 mV/s. A one compartment cell with a standard three-electrode set up was used, consisting of a 1 mm diameter glassy carbon disk as the working electrode, a platinum wire as the counter electrode and a silver wire as the pseudo-reference electrode, in a solution of anhydrous *o*-DCB containing 0.05 M *n*-Bu_4NPF_6. Ferrocene was added to the solution at the end of each experiment as an internal standard.

Synthetic procedures

Scheme S-1. Synthesis of diynes S-3a-c.

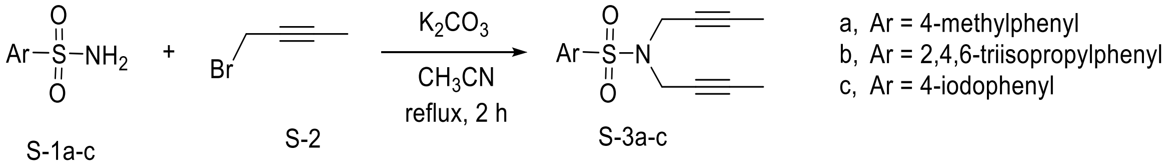

In a round-bottom flask equipped with a reflux condenser and a magnetic stirrer, a suspension of the corresponding sulfonamide (1 eq) and K_2CO_3 (5 eq) was stirred in acetonitrile and heated to 80 ºC. 1-bromo-2-butyne (2.1 eq) was added dropwise to the reaction mixture and stirred at 80 ºC for 2 h until completion (TLC monitoring). The reaction mixture was allowed to cool to room temperature, the solids were filtered off and the filtrate was concentrated to dryness and purified by column chromatography.

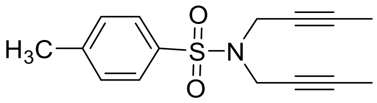

Diyne S-3a was obtained from p-toluenesulfonamide (1 g, 5.84 mmol) following the general procedure. Purification by column chromatography (SiO_2, 40–63 µm, CH_2Cl_2/hexanes 1:1) afforded diyne **S-3a** (1.49 g, 93% yield) as a pale yellow solid. Spectral data in accordance with literature values.[1]

MW ($C_{15}H_{17}NO_2S$): 275.36 g/mol; **Rf**: 0.35 (CH_2Cl_2/hexanes 1:1); **^{1}H NMR (400 MHz, $CDCl_3$) (ppm)**: 1.64 (t, *J* = 2.3 Hz, 6H), 2.41 (s, 3H), 4.06 (q, *J* = 2.3 Hz, 4H), 7.28 (d, *J* = 7.9 Hz, 2H), 7.71 (d, *J* = 8.3 Hz, 2H).

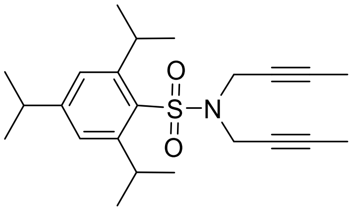

Diyne S-3b was obtained from 2,4,6-triisopropylbenzensulfonamide **S-1b** (2.00 g, 7.05 mmol), following the general procedure. Purification by column chromatography (SiO_2, 40–63 µm, hexanes/AcOEt 20:1) afforded compound **S-3b** (2.32 g, 85% yield) as a colorless solid. Spectral data in accordance with literature values.[1]

MW ($C_{23}H_{33}NO_2S$): 387.58 g/mol; **Rf**: 0.40 (CH_2Cl_2/hexanes 1:1); **^{1}H NMR (400 MHz, $CDCl_3$) (ppm)**: 1.24 (d, *J* = 6.8 Hz, 12H), 1.25 (d, *J* = 6.8 Hz, 6H), 1.78 (t, *J* = 2.2 Hz, 6H), 2.89 (sept, *J* = 6.8 Hz, 1H), 4.04 (q, *J* = 2.2 Hz, 4H), 4.10 (sept, *J* = 6.8 Hz, 2H), 7.15 (s, 2H).

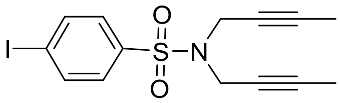

Diyne S-3c was obtained from 4-iodobenzenesulfonamide (2.00 g, 7.07 mmol) following the general procedure. Purification by column chromatography (SiO2, 40–63 µm, CH_2Cl_2/hexanes 1:1) afforded compound **S-3c** (2.55 g, 93% yield) as a pale yellow solid. Spectral data in accordance with literature values.[1]

MW ($C_{14}H_{14}INO_2S$)**:** 387.24 g/mol**;** Rf: 0.44 (CH_2Cl_2/hexanes 1:1)**; 1H NMR (400 MHz, $CDCl_3$) (ppm):** 1.65 (t, *J* = 2.4 Hz, 6H), 4.06 (q, *J* = 2.4 Hz, 4H), 7.53 (d, *J* = 8.8 Hz, 2H), 7.85 (d, *J* = 8.8 Hz, 2H)**.**

Scheme S-2. Synthesis of *bis*-(fulleroids) 1a-b, S-5.

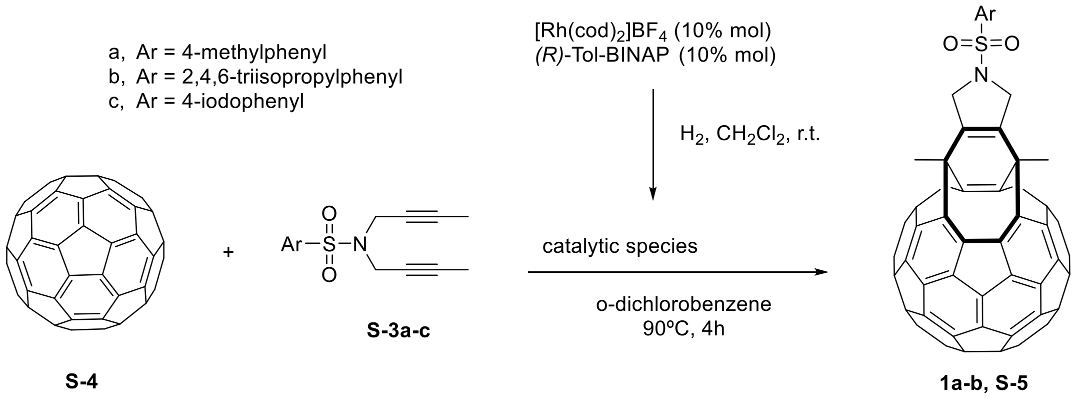

In a 10 mL capped vial in an inert atmosphere, a solution of [Rh(cod)$_2$]BF$_4$ (0.1 equivalents) and *(R)*-Tol-BINAP (0.1 equivalents) in anhydrous CH_2Cl_2 (4 mL) was prepared. Hydrogen gas was bubbled into the catalyst solution for 30 min before it was concentrated to dryness, dissolved in anhydrous *o*-dichlorobenzene and introduced into a solution of C_{60} (1 equivalent) and the corresponding diyne **S-3a-c** (5 equivalents) in anhydrous *o*-dichlorobenzene (1.4 mM), preheated to 90 °C. The resulting mixture was stirred at 90 °C for 4 h. It was then allowed to cool down to room temperature, concentrated to dryness and purified by column chromatography.

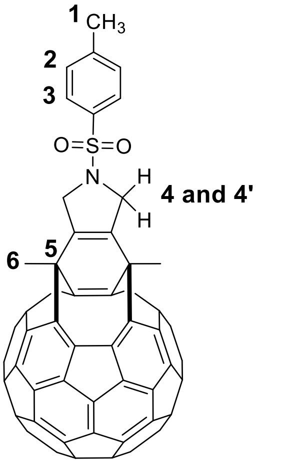

1a was obtained from C_{60} (50 mg, 0.07 mmol) and diyne **S-3a** (96 mg, 0.35 mmol), following the general procedure. Purification by column chromatography (SiO_2, 40–63 µm, toluene) afforded **1a** (36 mg, 52% yield) as a dark brown solid. The analytical samples were prepared by washing **1a** with CH_3OH (3 x 2 mL) and *n*-pentane (3 x 2 mL).

MW ($C_{75}H_{17}NO_2S$): 996.03 g/mol; **Rf**: 0.66 (CH_2Cl_2); **^{1}H NMR (400 MHz, CDCl$_3$) (ppm):** 2.36 (s, 3H, H**1**), 2.79 (s, 6H, H**6**), 4.42-4.50 (m, 2H, H**4**), 4.50-4.62 (m, 2H, H**4'**), 7.34 (d, *J* = 8.0 Hz, 2H, H**2**), 7.79 (d, *J* = 8.0 Hz, 2H, H**3**). Spectral data in accordance with literature values.[1]

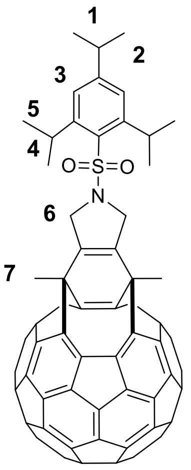

1b was obtained from C_{60} (50 mg, 0.07 mmol) and diyne **S-3b** (136 mg, 0.35 mmol), following the general procedure. Purification by column chromatography (SiO_2, 40–63 μm, toluene) afforded **1b** (34 mg, 44% yield) as a dark brown solid. The analytical samples were prepared by washing with and CH_3OH (3 x 2 mL) and *n*-pentane (3 x 2 mL).

MW ($C_{83}H_{33}NO_2S$): 1108.24 g/mol; **Rf**: 0.84 (CH_2Cl_2); **^{1}H NMR (400 MHz, $CDCl_3$) (ppm)**: 1.27 (d, *J* = 6.8 Hz, 6H, H**1**), 1.31 (d, *J* = 6.8 Hz, 12H, H**5**), 2.83 (s, 6H, H**7**), 2.93 (m, 1H, H**2**), 4.25-4.32 (m, 4H, H**6**), 4.67-4.72 (m, 2H, H**4**), 7.21 (s, 2H, H**3**). Spectral data in accordance with literature values.[1]

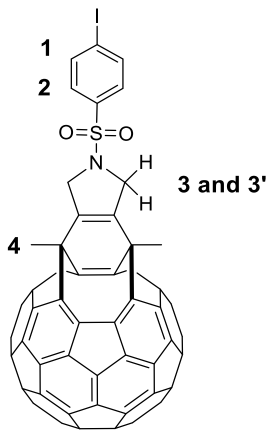

S-5 was obtained from C_{60} (100 mg, 0.14 mmol) and diyne **S-3c** (135 mg, 0.35 mmol), following the general procedure. Purification by column chromatography (SiO_2, 40–63 μm, toluene) afforded **S-5** (60 mg, 39% yield) as a dark brown solid. The analytical samples were prepared by washing with CH_3OH (3 x 2 mL) and *n*-pentane (3 x 2 mL).

MW ($C_{74}H_{14}IO_2NS$): 1107.90 g/mol; **Rf**: 0.74; (CH_2Cl_2); **^{1}H NMR: (400 MHz, *o*-DCB-d_4/CS_2) (ppm)**: (s, 2.61, 3H, H**4**), 4.31-4.41 (m, 2H, H**3**), 4.42-4.47 (m, 2H, H**3'**), 7.66 (d, *J* = 8.5 Hz, 2H, H**2**), 7.79 (d, *J* = 8.5 Hz, 2H, H**1**). Spectral data in accordance with literature values.[1]

Scheme S-3. Synthesis of *bis*-(fulleroid) 1c.

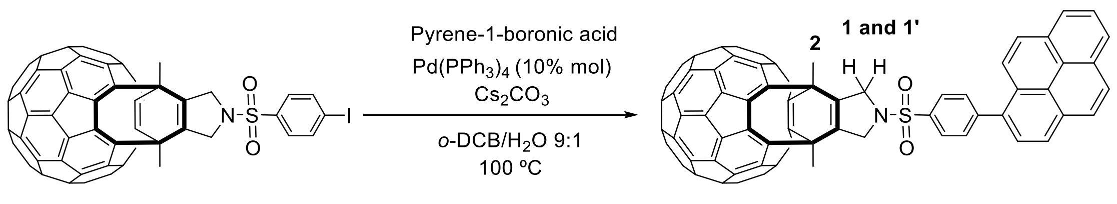

S-5 **1c**

In a two-necked round-bottom flask, equipped with a reflux condenser and a magnetic stirrer, a suspension of iodo *bis*-fulleroid derivative **S-5** (60 mg, 0.054 mmol), pyrene-1-boronic acid (27 mg, 0.11 mmol), $CsCO_3$ (46 mg, 0.14 mmol) and $Pd(PPh_3)_4$ (5.8 mg, 0.005 mmol) was prepared in a mixture of *o*-dichlorobenzene and water (9:1, 5.4 mM) under a stream of nitrogen gas. The resulting mixture was heated at 100 ºC and stirred in an inert atmosphere for 4 h (TLC monitoring). The reaction mixture was then allowed to cool to room temperature, concentrated to dryness and purified by column chromatography (SiO_2, 40–63 µm, CH_2Cl_2/hexanes 7:3) to afford **1c** (49 mg, 78% yield) as a dark brown solid. The analytical samples were prepared by washing with CH_3OH (3 x 2 mL) and *n*-pentane (3 x 2 mL).

MW ($C_{90}H_{23}NO_2S$): 1182.24 g/mol; **Rf**: 0.72 (CH_2Cl_2); **^{1}H NMR (400 MHz, $CDCl_3$) (ppm)**: 2.86 (s, 6H, H**2**), 457-4.65 (m, 2H, H**1**), 4.70-4.76 (m, 2H, H**1'**), 7.83-8.24 (m, 13H, H**Ar**). Spectral data in accordance with literature values.[1]

Scheme S-4. Synthesis of dicarbonyl C_{60} derivatives 2a-c.

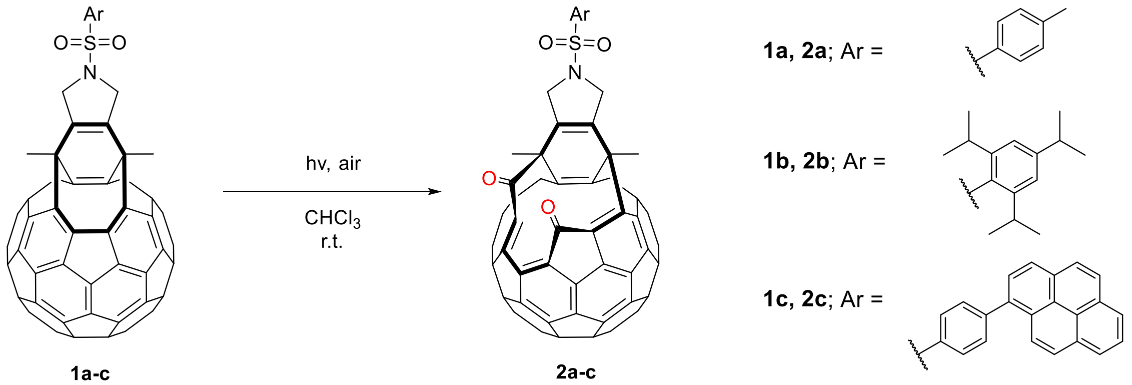

1a-c **2a-c**

A solution of the corresponding C_{60} derivative **1a-c** in $CHCl_3$ (5 mL) was prepared in a vial and exposed to sunlight and air for 5 h. The resulting solution was concentrated under reduced pressure and purified by column chromatography to afford the corresponding dicarbonylic C_{60} derivative **2a-c** as a dark brown solid.

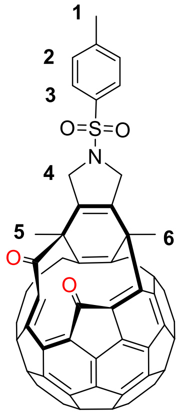

2a was obtained from **1a** (71 mg, 0.07 mmol) following the general procedure. Purification by column chromatography (silica gel, 40–63 μm, hexanes/CH_2Cl_2) afforded **2a** (66 mg, 92%) as a dark brown solid. The analytical samples were prepared by washing with CH_3OH (3 x 2 mL) and *n*-pentane (3 x 2 mL).

MW ($C_{75}H_{17}NO_4S$): 1028.02 g/mol; **Rf**: 0.40 (CH_2Cl_2); **^{1}H NMR (400 MHz, $CDCl_3$) (ppm)**: 2.21 (s, 3H, H**5**), 2.46 (s, 3H, H**1**), 2.57 (s, 3H, H**6**), 4.90-5.00 (m, 3H, H**4**), 5.51-5.56 (m, 1H, H**4′**), 7.47 (d, *J* = 8.0 Hz, 2H, H**2**), 8.10 (d, *J* = 8.4 Hz, 2H, H**3**). Spectral data in accordance with literature values.[1]

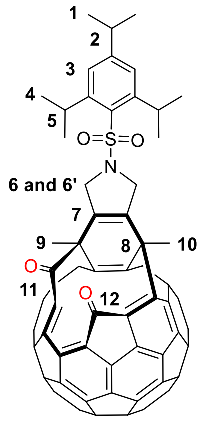

2b was obtained from **1b** (34 mg, 0.031 mmol) following the general procedure. Purification by column chromatography (SiO_2, 40–63 μm, hexanes/CH_2Cl_2 1:1) afforded **2b** (31 mg, 89% yield) as a dark brown solid. The analytical samples were prepared by washing with CH_3OH (3 x 2 mL) and *n*-pentane (3 x 2 mL).

MW ($C_{83}H_{33}NO_4S$): 1140.24 g/mol; **Rf**: 0.80 (CH_2Cl_2); **IR (ATR) *ν* (cm^{-1})**: 2925, 1740, 1687 1318, 1152; **^{1}H NMR (400 MHz, $CDCl_3$) (ppm)**: 1.36 (d, *J* = 6.8, 6H, H**1**), 1.45 (d, *J* = 6.8, 6H, H**4**), 1.46 (d, *J* = 6.8, 6H, H**4′**), 2.26 (s, 3H, H**9**), 2.64 (s, 3H, H**10**), 3.02 (sept, *J* = 6.8, 1H, H**2**), 4.62 (sept, *J* = 6.8, 2H, H**5**), 4.93-5.04 (m, 3H, H**6**), 5.56-5.51 (m, 1H, H**6′**), 7.34 (s, 2H, H**3**); **^{13}C NMR (101 MHz, $CDCl_3$) (ppm)**: 23.8 (C**1**), 25.3 (C**4**), 29.9 (C**5**), 31.8 (C**9)**, 32.0 (C**10**), 34.4 (C**2**), 42.9 (C**8)**, 51.8 (C**8′**), 53.9 (C**6**), 55.3 (C**6′**), 124.2 (C**3**), 131.0, 131.4, 131.5, 131.8, 132.4, 133.3, 133.4, 134.7, 135.8, 136.0, 136.2, 136.3, 136.4, 136.9, 137.7, 138.0, 139.1, 139.9, 140.0, 140.3, 140.3, 140.5, 140.6, 140.7, 141.1, 141.3, 141.9, 142.1, 142.5, 142.9, 143.0, 143.1, 144.2, 144.3, 144.6, 144.8, 145.2, 145.3, 145.5, 145.6, 145.8, 145.9, 145.9, 145.9, 146.0, 146.0, 146.2, 146.2, 146.4, 146.6, 147.1, 147.6, 147.9, 147.9, 147.9, 148.9, 150.1, 150.3, 151.7, 153.2, 154.6, 191.6, 201.7 (two overlapping carbons); **UV-vis ($CHCl_3$) max:** 421, 450, 510, 610 and 670 nm; **ESI-HRMS (*m*/*z*)** calcd for $[M+Na]^+$ = 1162.2028; found 1162.2005.

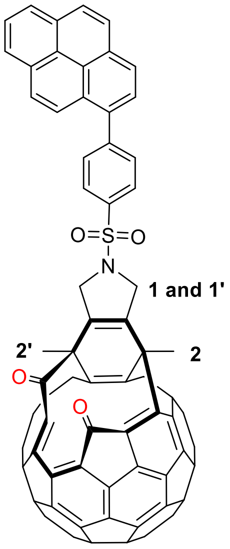

2c was obtained from **1c** (49 mg, 0.041 mmol) following the general procedure. Purification by column chromatography (silica gel, 40–63 µm, hexanes/CH_2Cl_2) afforded **2c** (49 mg, 98% yield) as a dark brown solid. The analytical samples were prepared by washing with CH_3OH (3 x 2 mL) and *n*-pentane (3 x 2 mL).

MW ($C_{90}H_{23}NO_4S$): 1214.24 g/mol; **Rf**: 0.50 (CH_2Cl_2); **IR (ATR)** ν **(cm^{-1})**: 2916, 1727, 1372, 1157; **^{1}H NMR (400 MHz, $CDCl_3$) (ppm)**: 2.27 (s, 3H, H**2**), 2.64 (s, 3H, H**2'**), 5.00 – 5.11 (m, 3H, H**1**), 5.65 – 5.72 (m, 1H, H**1'**), 7.80 (d, *J* = 9.2 Hz, 1H, H**Ar**), 7.93 – 8.21 (m, 10H, H**Ar**) 8.45 (d, *J* = 7.9 Hz, 2H, H**Ar**); **UV-vis ($CHCl_3$)** $_{max}$**:** 421, 450, 510, 610 and 670 nm; **: ESI-HRMS (*m*/*z*)** calcd for $[M+Na]^+$ = 1236.1245; found 1236.1240.

1. H- and ^{13}C-NMR spectra

Diyne S-3a

Figure S-1. ^{1}H NMR (400 MHz, $CDCl_3$).

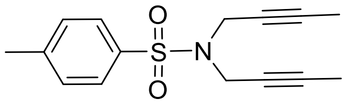

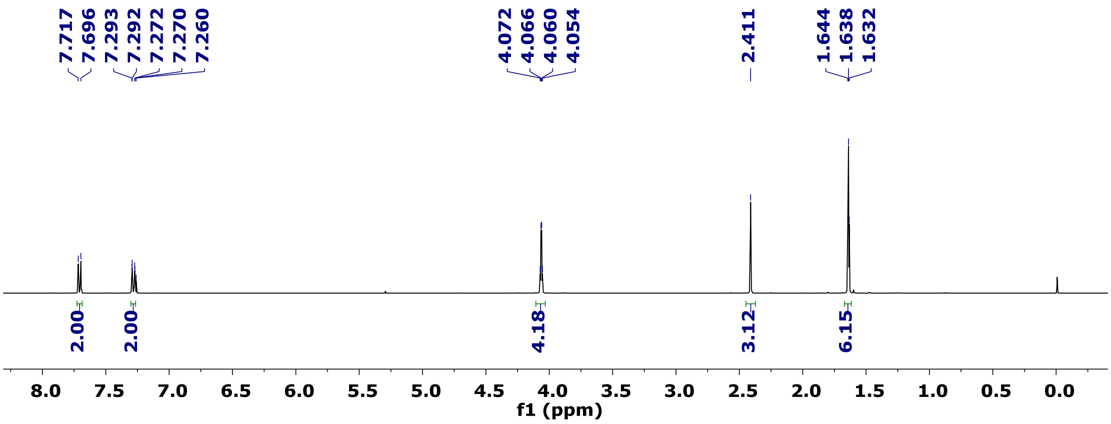

Diyne S-3b

Figure S-2. ^{1}H NMR (400 MHz, $CDCl_3$).

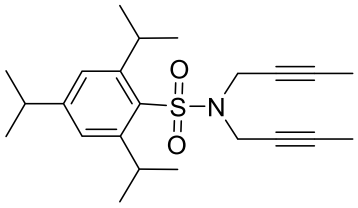

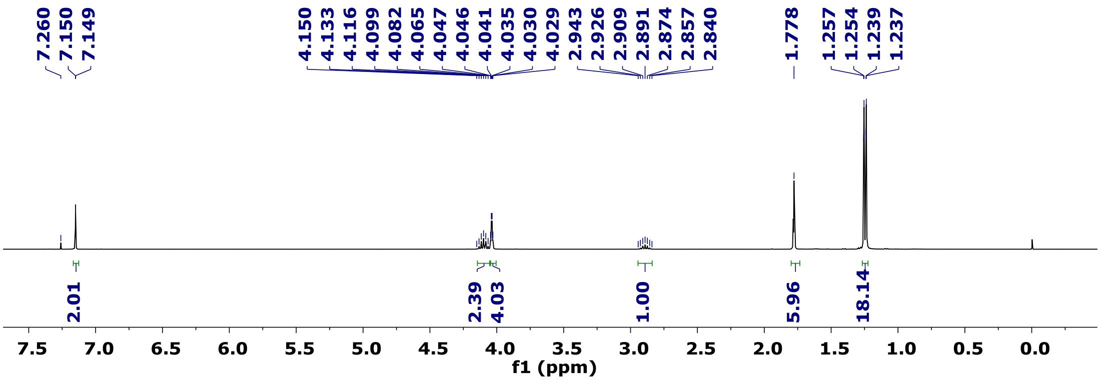

Diyne S-3c

Figure S-3. ¹H NMR (400 MHz, $CDCl_3$).

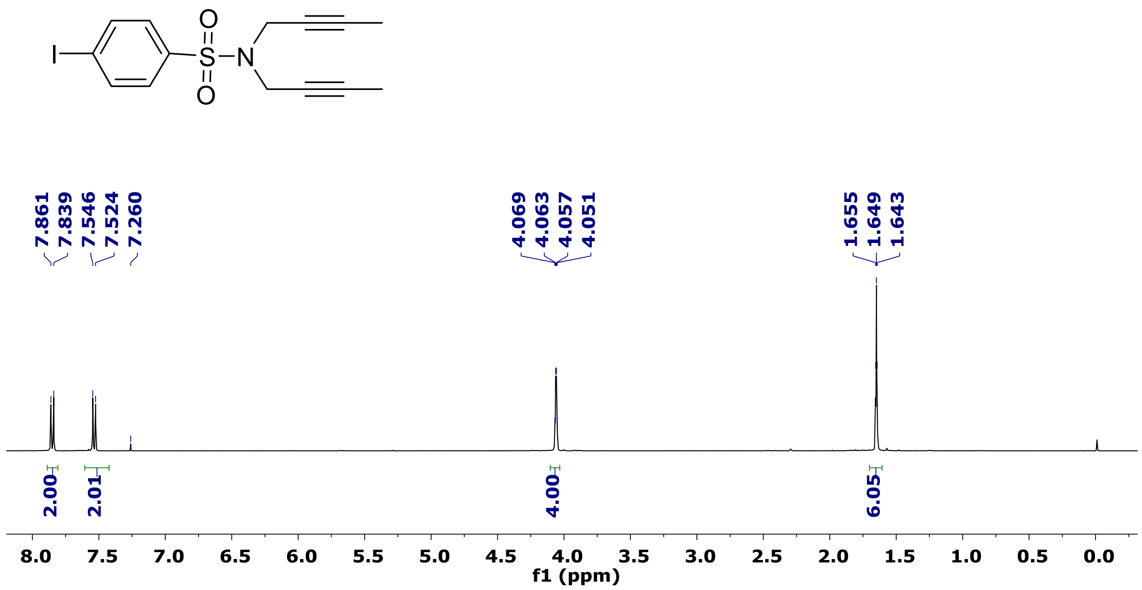

Compound 1a

Figure S-4. ¹H NMR (400 MHz, $CDCl_3$).

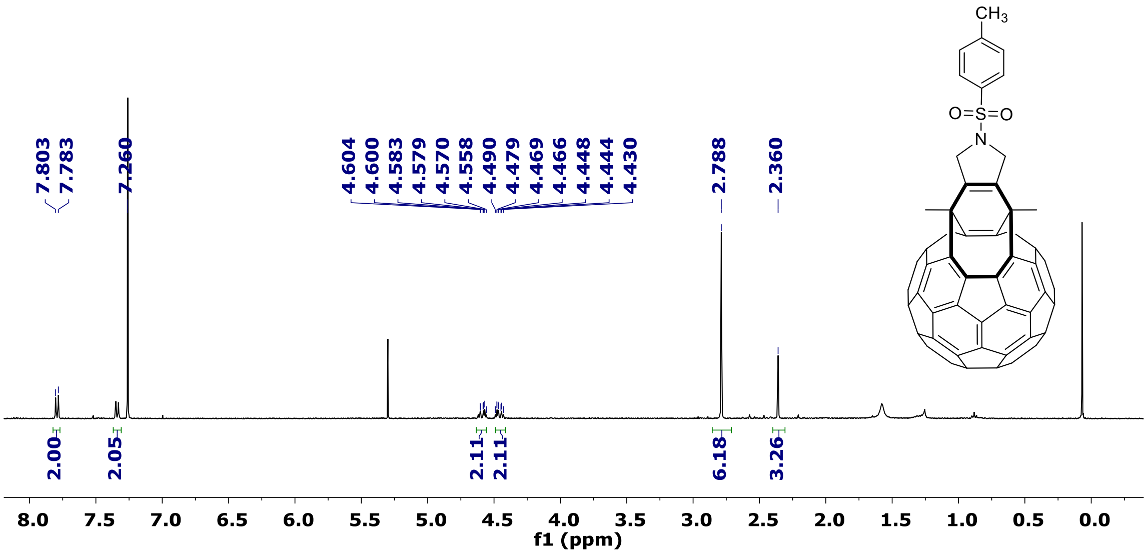

Compound 1b

Figure S-5. ¹H NMR (400 MHz, $CDCl_3$).

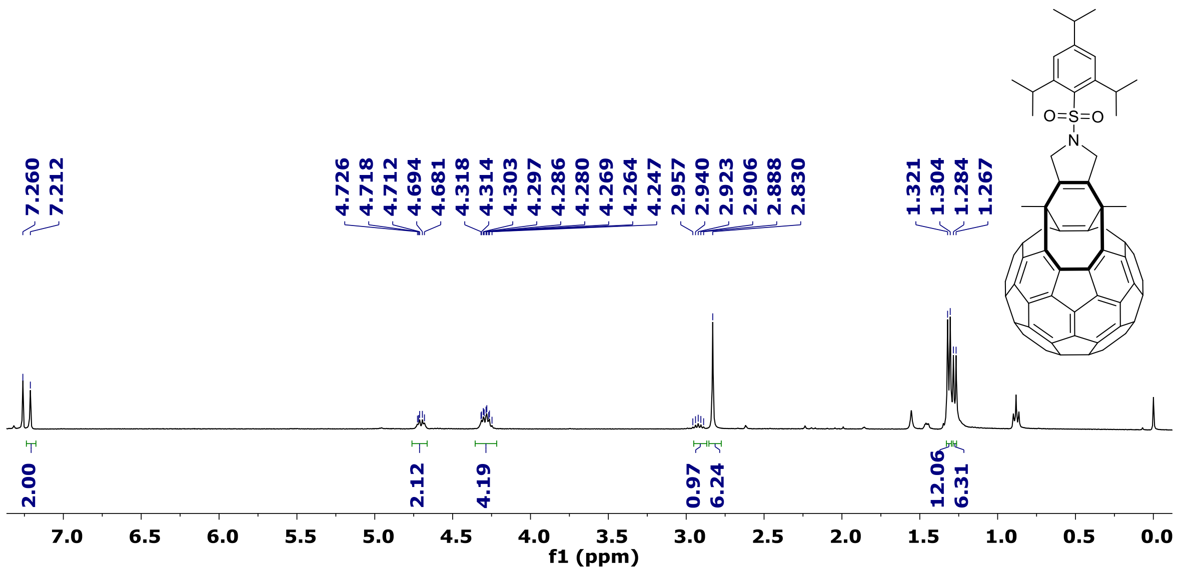

Compound S-5

Figure S-6. ¹H NMR (400 MHz, o-DCB-*d4*/CS_2).

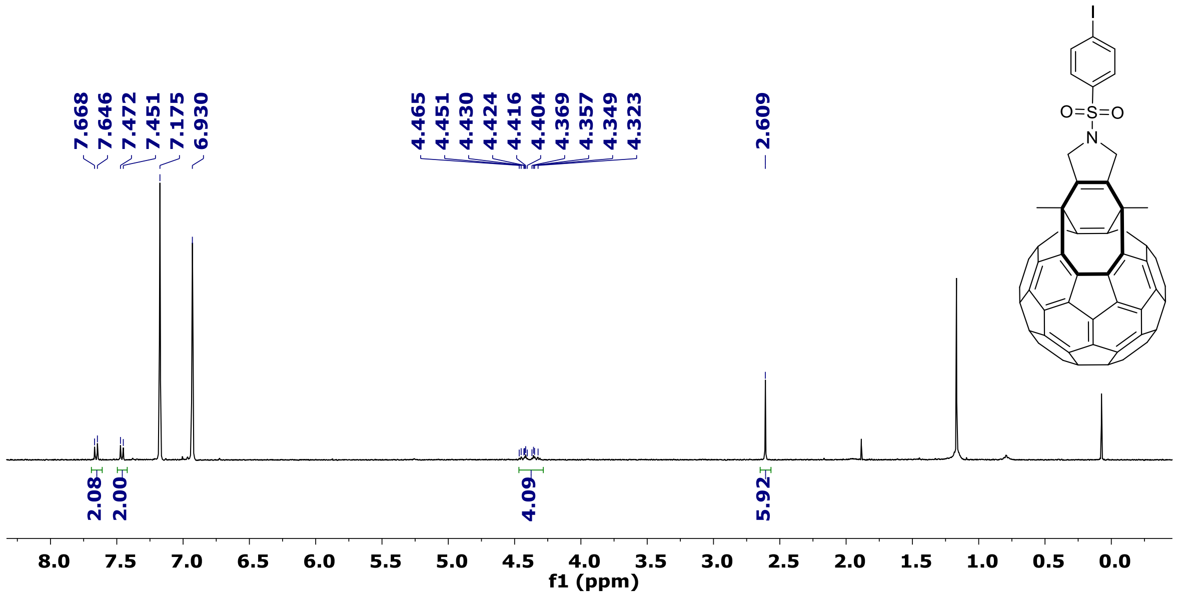

Compound 1c

Figure S-7. ¹H NMR (400 MHz, $CDCl_3$).

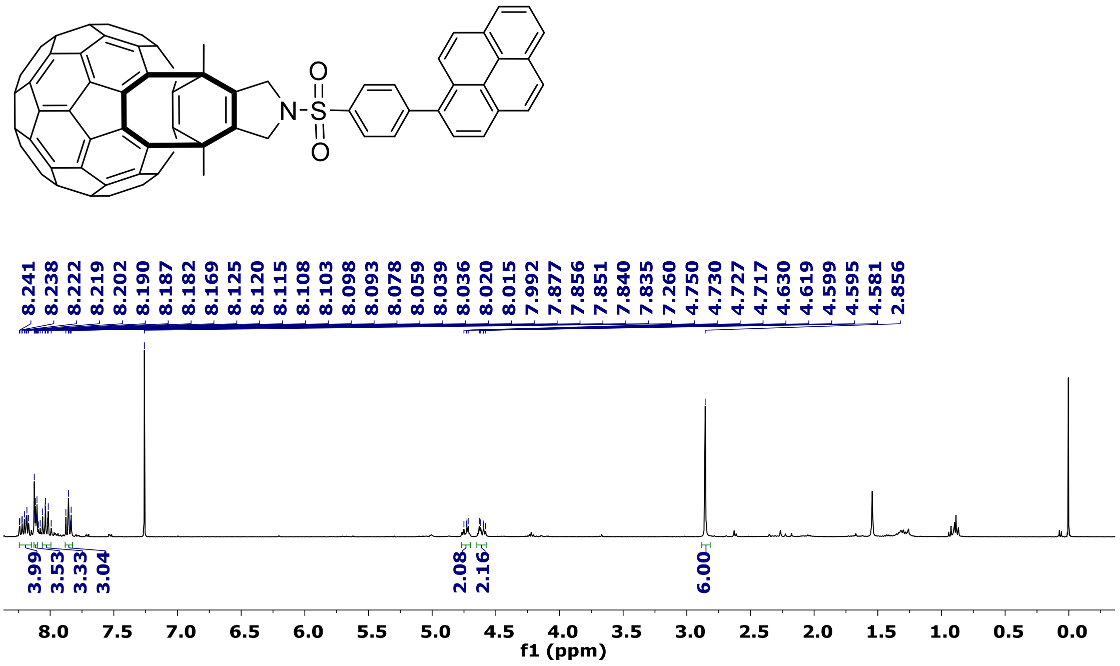

Compound 2a

Figure S-8. ¹H NMR (400 MHz, $CDCl_3$).

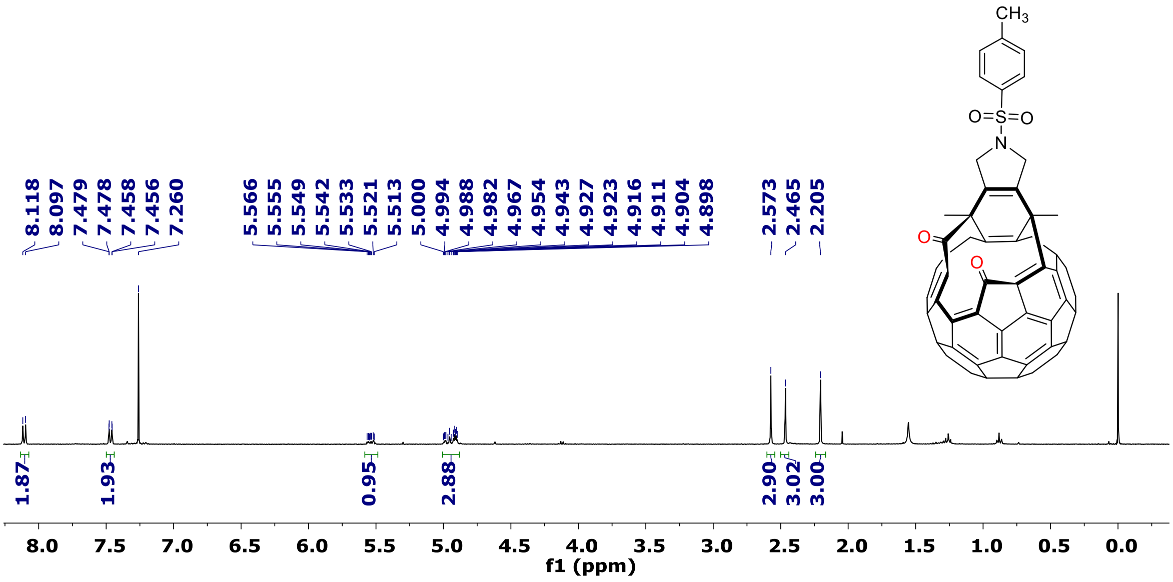

Compound 2b

Figure S-9. ^{1}H NMR (400 MHz, $CDCl_3$).

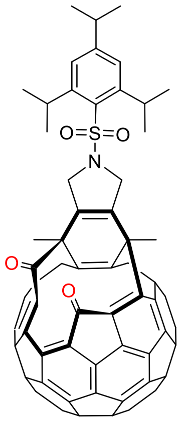

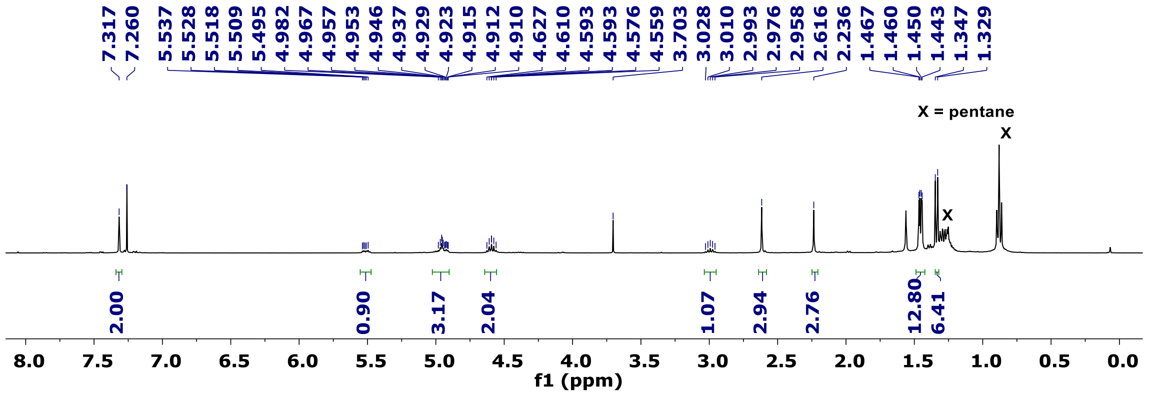

Figure S-10. ^{13}C NMR (100 MHz, $CDCl_3$).

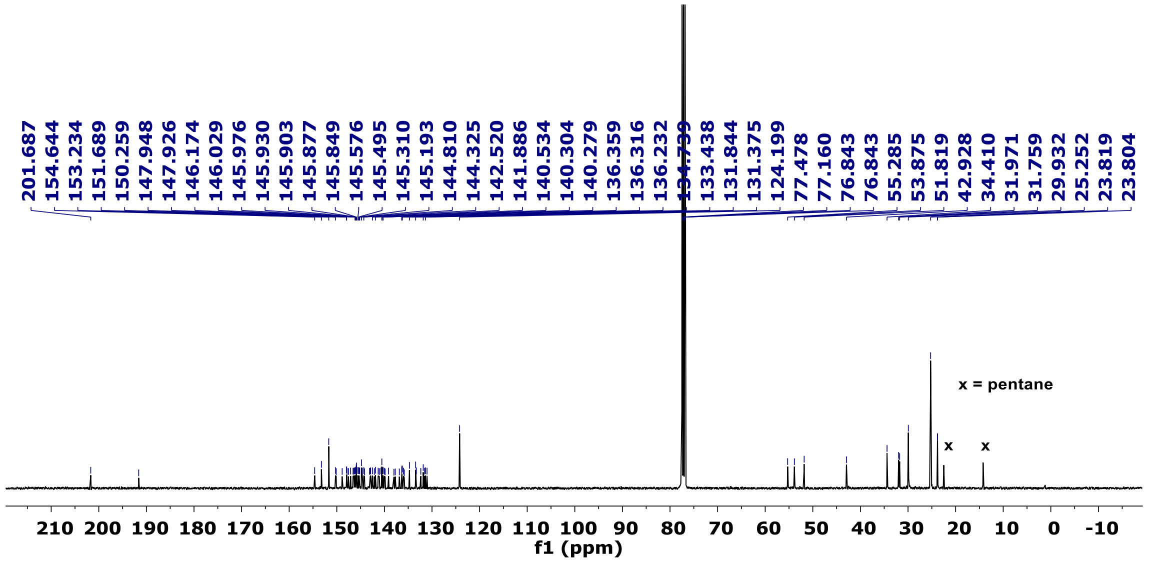

Compound 2c

Figure S-11. ^{1}H NMR (400 MHz, $CDCl_3$).

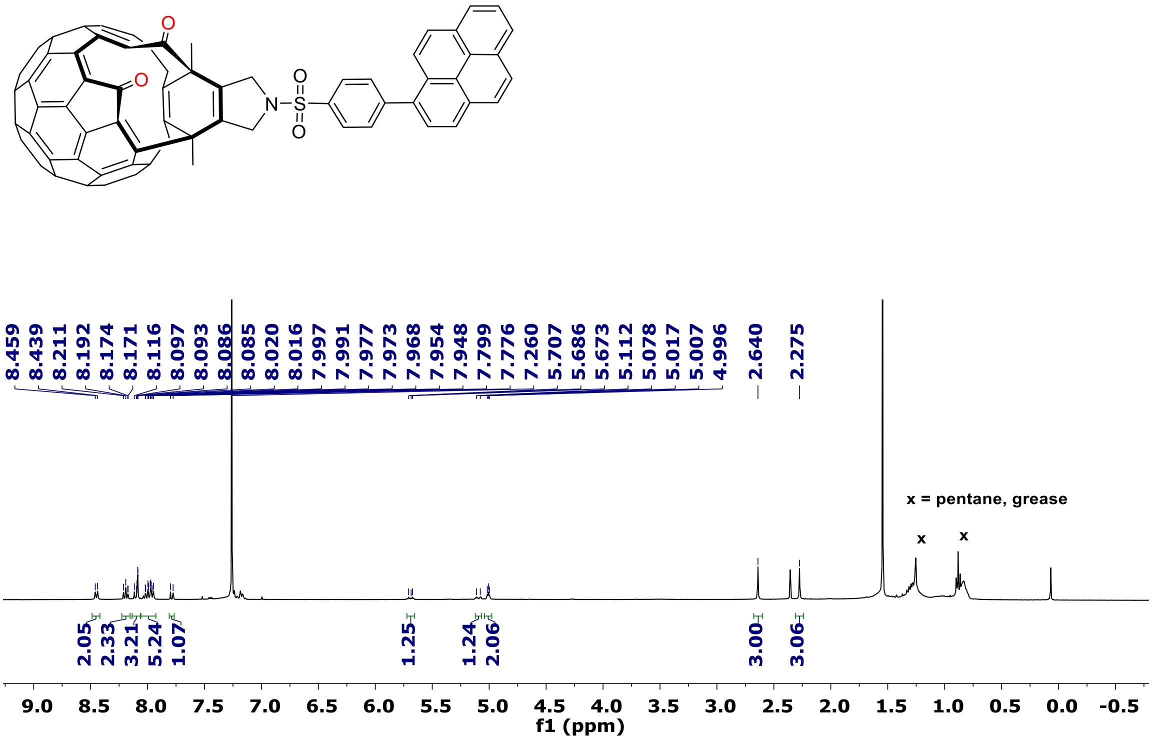

Figure S-12. UV-vis spectra of compounds 2a-c and $PC_{61}BM$.

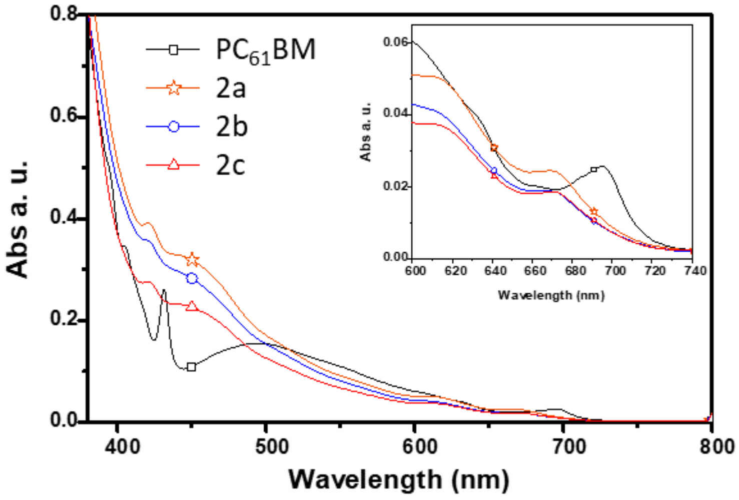

Figure S-13. Cyclic voltammetry of compounds 2a-c.

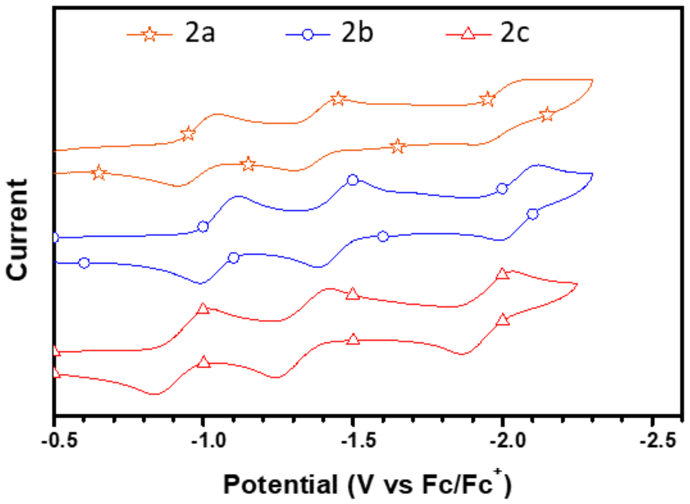

Figure S-14. Time-resolved photoluminescence of perovskite, perovskite/compounds 2a-c and perovskite/PC61BM films.

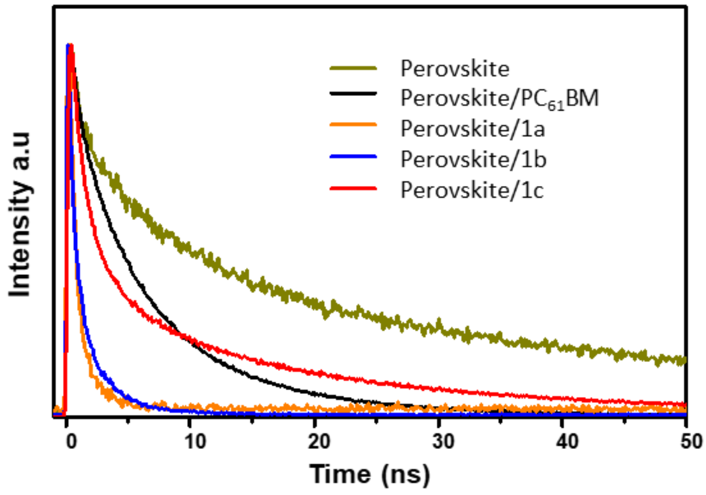

Figure S-15. Fullerene derivatives 2a-c and $PC_{61}BM$ in *o*-dichlorobezene (20 mg/mL).

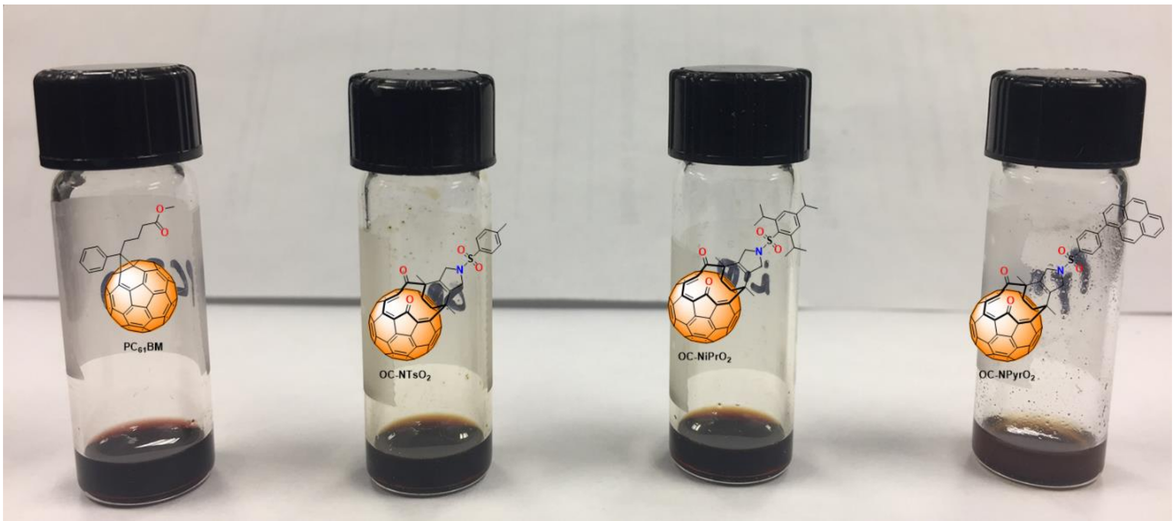

Figure S-16. *J-V* curves of the inverted PSCs based on $PC_{61}BM$ (a) and 2a,b (b and c, respectively) with respect to forward and reverse scan directions (the scanning rate was 100 mV/s).

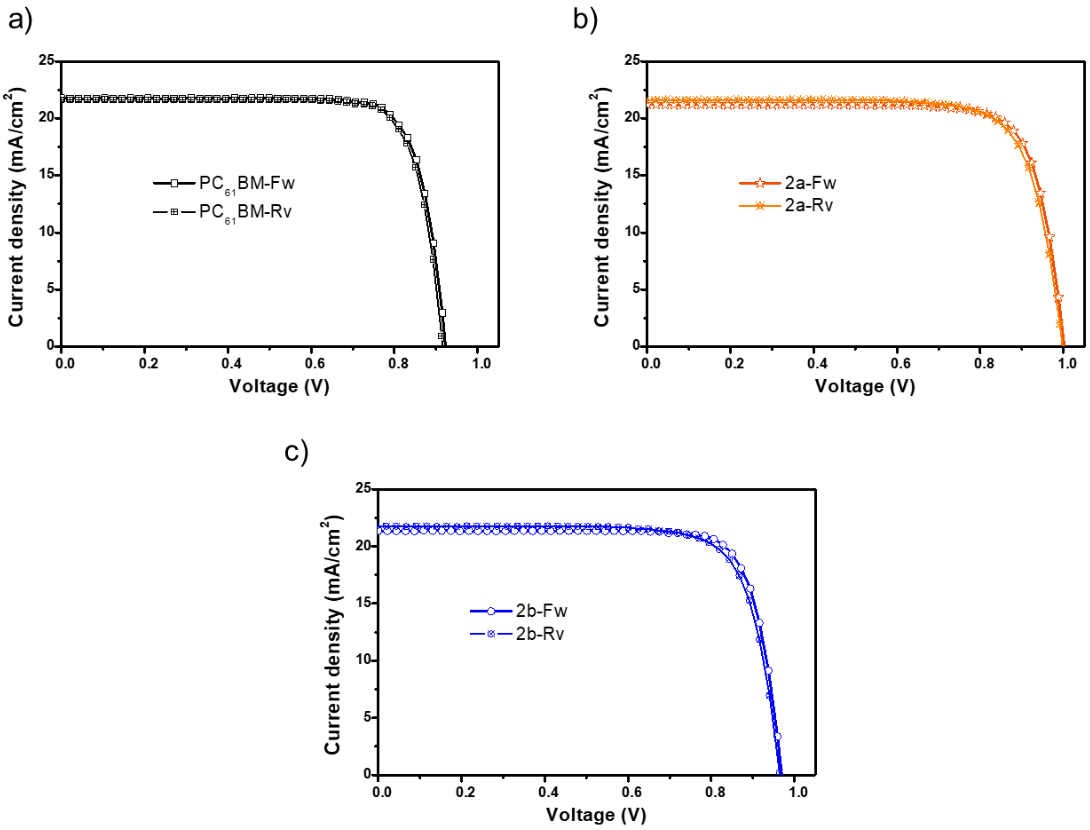

Figure S-17. EQE measurements for 2a-c and $PC_{61}BM$-based devices.

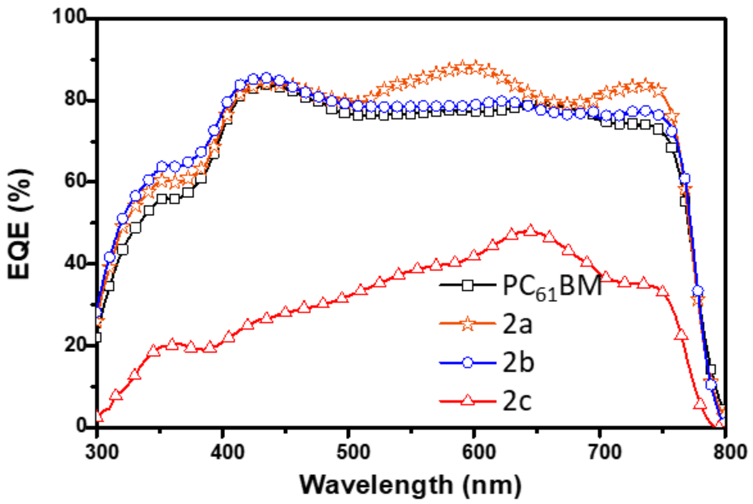

Figure S-18. Stability studies of 2a-c and $PC_{61}BM$-based devices.

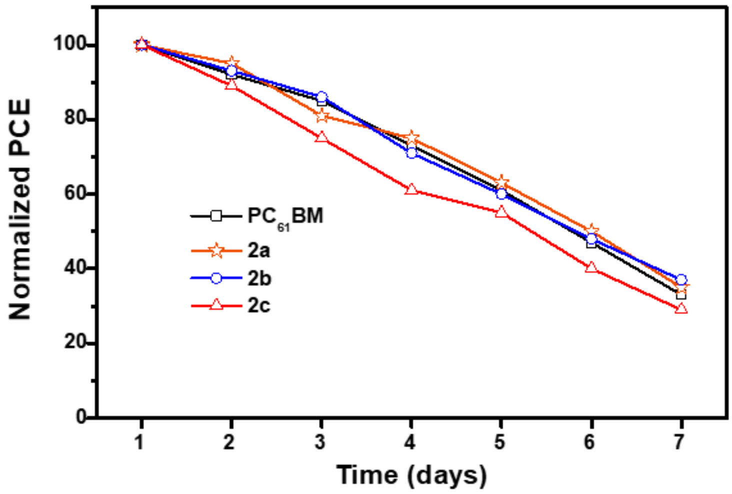

Device fabrication

$PC_{61}BM$ (99%) was bought from SES Research. Methylammonium iodide (CH_3NH_3I, 99.5%) was bought from Greatcellsolar. PbI_2 (99%) was bought from Sigma-Aldrich. The configuration used for the fabrication of the PSCs was ITO/PEDOT:PSS/$CH_3NH_3PbI_3$/ETM/Ag. The patterned ITO glass substrates were cleaned sequentially with detergent, deionized water and acetone, each step for 30 min, then dried with nitrogen gas and finally treated in a UV-ozone oven for 30 min. After passing through a 0.45 μm PVDF filter, the PEDOT:PSS solution (Baytron P VP AI 4083) was spin-coated onto

the treated ITO substrates at 5000 rpm for 30 s and heated at 150 °C for 15 min in air. Then the substrates were transferred to a N_2-filled glovebox where $CH_3NH_3PbI_3$ (1 M solution in DMF) was spin-coated on top of the PEDOT:PSS coated substrates at 800 rpm for 10 s and at 4000 rpm for 25 s. 80 μL of toluene were added 5 s after the second step and then the devices were annealed at 70 °C for 60 min. The top-view image (Figure S-19) shows a homogeneous perovskite layer with large crystalline grains (~400 nm) and without apparent pinholes. The fullerene derivatives dissolved in CB (20 mg/mL) were spin-coated onto the $CH_3NH_3PbI_3$ layer at 5000 rpm for 30 s. Finally, Ag electrodes (100 nm) were deposited by thermal evaporation under a pressure of $1{\times}10^{-6}$ Torr through a shadow mask. The active area of the fabricated devices was 6 mm^2. The Al electrodes were encapsulated with a UV-curable epoxy resin and a glass slide before testing. Stability studies were conducted on unencapsulated devices.

Device Characterization

J-V characteristics of photovoltaic cells were tested using a Keithley 2420 source meter under a Photo Emission Tech SS100 Solar Simulator, and the light intensity was calibrated by a standard Si solar cell. The EQE was measured using a Bentham (from Bentham Instruments Ltd) measurement system. The light intensity was calibrated using a single-crystal Si photovoltaic cell as reference. The *J-V* and EQE measurements were obtained in air. The SEM images were collected using a ZEISS Sigma FE-SEM, where the electron beam was accelerated in the range of 500 V to 30 kV. Film thicknesses were measured using a KLA Tencor profilometer. The steady-state PL spectra were recorded on a Horiba Yvon Nanolog spectrometer coupled with a time-correlated single photon counting (TCSPC) with nanoLED excitation sources for time-resolved emission measurements.

Figure S-19. Top-view SEM image of the perovskite film.

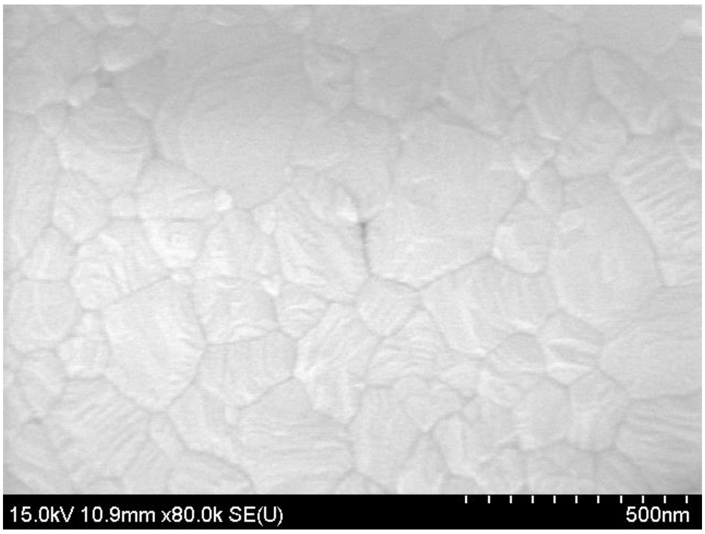

References

[1]A. Artigas, A. Pla-Quintana, A. Lledó, A. Roglans and M. Solà, *Chem. Eur. J.*, **2018**, *24*, 10653-10661.
